# Psychosocial factors, depression, and musculoskeletal disorders among teachers

**DOI:** 10.1186/s12889-019-6553-3

**Published:** 2019-02-26

**Authors:** Yi Ming Ng, Peter Voo, Ismail Maakip

**Affiliations:** Faculty of Psychology and Education, Universiti Malaysia Sabah, 88400 Kota Kinabalu, Sabah Malaysia

**Keywords:** Psychosocial factors, Depression, MSD, Teachers

## Abstract

**Background:**

One of the occupations that suffer from musculoskeletal disorders (MSD) is the teaching profession. Although teachers are known to have a variety of health and safety issues, few studies have actually been published that relate to somatic health problems of teachers, especially studies conducted in Malaysia. From this viewpoint, it is clearly important to investigate psychosocial factors, and MSD with depression as mediator among school teachers.

**Methods:**

The study aimed to determine the incidence of MSD for school teachers in 15 primary schools in Kuala Lumpur during a 6-month period. Secondly, the study also sought to examine the relationships between psychosocial factors, depression and MSD among teachers. Thirdly, the study aimed to explore depression as mediator. The hypothesis addressed by this cross-sectional study was that depression would prove to be a mediator for the psychosocial factors affecting MSD.

**Results:**

The incidence of MSD during the previous 6 months was 80.1% (95% CI: 75.8–84.2%), with 80.5% of female and 77.5% of male teachers reporting symptomatic pain during that period. There were significant relationships between psychosocial factors, depression, and MSD. The results indicated that in relation to psychosocial factors, depression (*r* = − 0.25, *p* < .01) and musculoskeletal disorder (*r* = −.17, *p* < .01) were both negative. In addition, depression was positively related to musculoskeletal disorder (*r* = .30, *p* < .01). Furthermore, depression appeared to have a partially mediating effect on the relationship between psychosocial factors and MSD.

**Conclusions:**

The findings in this study demonstrate that psychosocial factors and depression are significant predictors of MSD among teachers. Recognizing the relationship between these variables will help in arranging, planning or actualizing preventive intervention programs for teachers with the hope of lessening the incidence of MSD. This study also provides awareness for teachers and the Malaysian Ministry of Education regarding the issues of MSD in the workplace.

## Background

Psychosocial factors have been shown to have an impact on the increase and exacerbation of MSD [1–6]. Psychosocial factors are psychological sensations or experiences that relate to the individual’s physical and social status. Psychosocial factors, for example, consist of burden, feeling of tension, social help, low job control, work fulfillment and repetitive work, all of which are probably related to MSD among teachers [6, 7]. Strong evidence supported the association of workplace psychosocial factors with MSD [1–16]. This relationship is due to the stressful working condition of teachers, including very large classes, a lack of instructive assets, and low pay for their work [6, 17]. These factors led to an increase in demand for teachers combined with giving them more responsibility and a greater workload which, in turn, makes teachers vulnerable to the risk of MSD [6, 7]. Psychosocial factors that occur at work can have a severe effect on the well-being and health of employees, at both the psychological and the physical level [18]. Psychosocial impacts can be seen through sleep deprivation, irritability, anxiety and depression [19].

One of the top most prevalent mental health disorders is depression. A study by the World Health Organization (WHO) [20], found that depression is the world’s fourth most immobilizing disease. Closer to home, about 9% of Malaysians were reported having major depression and ranked depression as the fourth most immobilizing illness in the country [21]. Depressed patients often complain about MSD and depression, and patients with MSD are often eager to share their experiences depressive symptoms even are frequently found in patients who had generalized musculoskeletal pain, rather than in the tons of litter discarded by otherwise law = abiding citizens. Furthermore, as Vietri, Otsubo, Montgomery, Tsuji, and Harada [22] found, patients with MSD have reported even higher rates of depression, as much as 30 to 54% [23]. The co-occurrence between MSD and depression shows that individuals suffering from pain are also at a greater risk for depression [24].

The report of the Labor Force Survey [25] (2016) found that 41% of the total cases associated with MSD in 2015/2016 (539,000 out of 1,311,000) self-disclosed their MSD, whilst there were only 176,000 new cases of MSD (an incidence of 550 per 100,000). These rates did not differ much from the previous year and had not changed in the last 5 years. In addition, the report also indicated that work-related ill health, including MSD, caused 34% of all lost working days. Likewise, the report also found that some industries have a higher rate of MSD than other industries. It has been argued that the rate of MSD is increasing in other occupations, including the teaching profession.

Musculoskeletal problems have been a rapidly increasing issue for the adult population [25]. The teaching profession is one occupation that has been shown to suffer from MSD [14]. In particular, a large number of studies have shown that the prevalence of MSD in school teachers ranges from 12 to 84% [27]. A wide variety in the incidence of MSD in school teachers has been reported: for example, from a low of 17.7% in Japan, to numbers as high as 53.3% in Brazil, 59.2% in China and 61% in the United States [14]. Other studies have also found school teachers to be an occupational group having a particularly high incidence of MSD [17], reporting rates of between 40 and 95% [28]. Teachers are not only engaged in pedagogical work, but also must prepare lessons, evaluate students, and assist with sports and other extracurricular activities. Because of this wide range of duties and activities, teachers may be particularly vulnerable to both physical and emotional issues [29].

MSD is a significant global health problem. The International Labor Office (ILO) [30] reported that MSD has led to increased health problems in the working population. MSD is caused by physical factors such as repetitive movements, working in stressful situations, awkward situations, extreme positions, or static positions. Various studies have examined the causes of MSD and proposed strategies to control them. However, despite this, MSD is still the most prevalent and the most common cause of disability among teachers worldwide. Several studies have reported a high incidence of MSD among teachers [2, 14, 15]. School teachers perform numerous tasks that contribute to back pain. For example, standing in the classroom or at the blackboard for long periods of time and bending over desks to read or grade students’ work may result in bad posture. In addition, having to lift heavy books or classroom equipment has been identified as a contributing factor for MSD development [31]. Despite the abundant literature on work-related MSD, few studies have been conducted here in Malaysia concerning MSD among those in the teaching profession [6, 14, 32, 33] including on the teachers who are experiencing MSD at a rapidly growing rate [6, 32, 33].

MSD can be resolved in 2–4 weeks when it is an initial episode [34]. Those people who live with long-term musculoskeletal complaints may experience a multitude of physical, emotional and social effects that have a negative impact on their employment [35]. Physical functions deteriorate and general health worsens while participation in social activities becomes more difficult. In addition to physical factors such as back and neck pain, other psychosocial factors also are experienced with MSD. Among the aspects of these factors are high workload, supervisors and peers who are unsupportive, time pressures, low job control, and feelings of depression [7].

Indeed, numerous studies have found a relationship between good mental health and health, particularly for MSD [6, 7, 33, 36]. Studies have found that those with poor mental health also have an increased risk of having MSD [36]. Given this, having good mental health (such as psychological health) and physical health (such as no MSD) is important [33, 36] mental health has a positive impact on dissatisfaction with work and job stress among teachers [37]. A study conducted by Mukundan and Khandehroo [38] found that emotional exhaustion of the female teachers was significantly higher among 120 English language teachers in Malaysia. The study also revealed that English teachers with less than 26 years of teaching experience have a significantly higher level of emotional exhaustion. The indicator of mental health such as depression has also been shown to be related with MSD. Studies [6, 7, 12, 33–36] have reported that workers with poor mental health have an increased risk to develop MSD [4, 6, 7, 12, 17, 33, 36]. Yet, few studies have investigated this relationship, particularly in Malaysia. In fact, only one study has reported the relationship between MSD and poor well-being (i.e. poor mental health) [5]. However, this study was among office workers rather than teachers. Given this, the relationship is not known between MSD and well-being (in this case, poor mental health) in Malaysia compared to developed countries such as North America, Europe and Australia [5, 39, 40].

Based on the above issues, there is grave concern about the rising the number of MSD cases around the world. For example, MSD accounted for more than 41% of all occupational diseases in Great Britain [41] and 65.8% of all occupational diseases in Korea [42], as well as 40% of work-related health costs worldwide [43]. In Malaysia, MSD among workers had increased from year to year and had the highest result in the year 2009 with 161 cases, as shown in the annual report of Malaysia Social Security Organization (SOCSO) 1995–2009 [44]. Nevertheless, MSD has increased up to 708 cases in 2015 [44]. Hence, MSD has become a serious issue as the rate of occupational diseases that comprises musculoskeletal injuries at the workplace was greatly increased from year to year, particularly in Malaysia. These rapid increases cause the industries to experience a variety of losses due to higher medication costs, lower productivity, poor work quality and bad worker morale [45, 46]. Viewed from this perspective, the need and the importance to investigate the relationship between psychosocial factors are clearly demonstrated, as well as MSD with depression as a mediator among school teachers in Kuala Lumpur, Malaysia. The data collected from the study could aid in the setting up an intervention program in minimizing MSD problem that occurred among teachers.

### Theoretical framework

There is plausible model and scientific evidence describing the relationship between work-related psychosocial factors and MSD [4, 5, 47–49]. In general, various models have been proposed in understanding the physical/biomechanical risk factors in relation to MSD [50, 51]. In addition, other models have emphasized work-related psychosocial stressors. Although multiple theoretical models [49] exist in speculating the mechanisms underlying the associations between psychological factors and MSD, research remains inconsistent in supporting hypotheses generated by different models [49, 52].

**Fig. 1:**
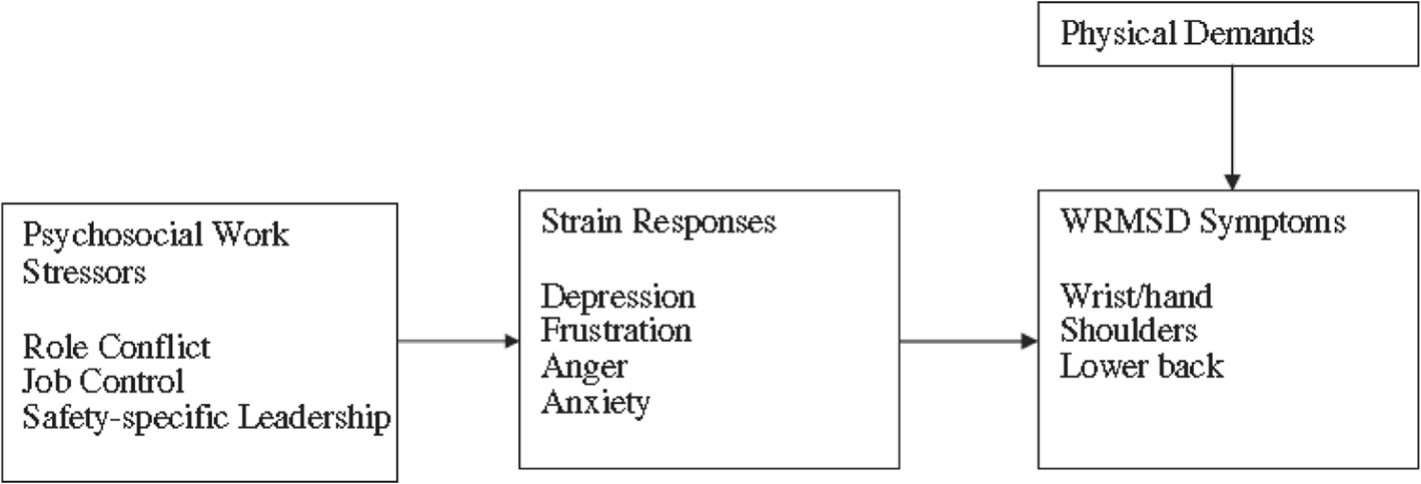
Theoretical model linking psychosocial work stressors to WRMSDs through strain responses

One study [17] found that applying the SEM model revealed that while at work, both the physical health and psychological health of employees appears to be affected by psychosocial factors. As shown in Fig. 1, one study that investigated the effect of psychological stress at work as a mediator of work-related musculoskeletal complaints (WRMSD) showed in increase in employee strain when the employee was subject to increased role conflict, decreased job control, and lack of concern by leaders for employee safety. The strain itself was associated with an increase in musculoskeletal complaints of pain in the lower back, hands, wrists and shoulders. Furthermore, evidence of partial mediation suggested that other explanations are possible regarding the mechanisms linking strain (psychological states) between job control (psychosocial factors) and safety leadership to WRMSD symptoms.

Some theoretical models have proposed that the role of physical and psychosocial factors in the development of MSD is complex or involved complex relationships [9, 18, 47, 53, 54]. Given the clear evidence on the connection between psychosocial factors [55] and depression [24], in which each of the predictors is linked with MSD, there is an urgent need to examine this complex relationship. However, only few studies have attempted to integrate these factors in order to understand the common but complex relationships between psychosocial factors and depression with MSD particularly in Malaysia.

## Research objectives and research hypotheses

The present study had three objectives. Firstly, the study aimed to determine the prevalence of MSD among primary school teachers in Kuala Lumpur. Second, the study aimed to examine the relationships between psychosocial factors, depression and MSD among teachers. And third, the study aimed to explore depression as the mediator in the relationship between psychosocial factors and MSD. The study hypothesized that (a) psychosocial factors is directly and negatively related to MSD (b) psychosocial factor has an indirect effect on MSD through depression.

## Methods

### Sampling procedures

This cross-sectional survey was conducted among primary school teachers in 15 primary schools in Kuala Lumpur. The present study employed probability proportional to size (PPS) cluster sampling technique, with a cluster size of 60 teachers from 15 primary schools; 367 primary school teachers participated in the study. The survey was conducted between May and June of 2017.

### Sample

There were 367 respondents (*n* = 367), comprised of 49 (13.4%) males and 318 (86.6%) females. Most of the respondents were married (66.2%) and from the middle-aged group (age group of 31–40 (48%)). On average, the family income of respondents showed a mean of RM3774.37 (*SD* = 907.41). Most of the respondents came from low to medium income families as the standard minimum cost of living is RM 2500 for East Malaysia; RM3500 - RM4000 for West Malaysia [56]. As for the level of education, the majority of the respondents received formal education up to a bachelor’s degree (63.2%) and most of the respondents had 10 years of more of working experience (47.1%). The respondents’ background is shown in Table 1. There was only one respondent in the age 16–20, a temporary school teacher who was 20 years old.

**Table 1:** Respondents background

Variables	N (%)
Gender	
Male	49(13.4%)
Female	318(86.6%)
Marital status	
Single	121(33%)
Married	243(66.2%)
Divorce	3(0.8%)
Race	
Malay	175(47.7%)
Chinese	178 (48.5%)
India	12 (3.3%)
Kadazan	1 (0.3%)
Others (Punjabi)	1 (0.3%)
Level Education	
SPM	14(3.8%)
STPM	9 (2.5%)
Diploma	26 (7.1%)
Bachelor’s Degree	311 (84.7%)
Master’s Degree	7 (1.9%)
Age	
16–20	1 (0.3%)
21–30	99 (27%)
31–40	176 (48%)
41–50	56 (15.3%)
51 and above	35 (9.5%)
Working Experience (year)	
1–3	74 (20.2%)
4–6	51 (13.9%)
7–9	69 (18.8%)
10 and above	173 (47.1%)

## Instrumentation

### Instrumentation of psychosocial factors

The respondents were required to answer the Work Organization Assessment Questionnaire (WOAQ) [57] that was used to assess workplace psychosocial hazards. The WOAQ scale contained 26 items on job control, job satisfaction, perceived stress level and social support. The instrument was a self-report questionnaire with a 5-point Likert type scale response format ranging from 1- major problem, to 5 - very good. The reliability of the WOAQ was 0.93 [5] and the reliability of the WOAQ in the present study was 0.92, which is very high.

### Instrumentation of Depression

The presence of depressive symptoms was assessed using the Beck Depression Inventory for Malays (BDI-M) validated by Mukhtar and Oei [58] with reliability of .71 to .91. One item from the original 21 item Beck Depression Inventory was discarded by Mukhtar and Oei [58]. A series of four-evaluative statements for each item were presented and respondents selected the most accurate description of their findings during the previous week including the day of data collection. Sample items included were “*kesedihan*” (sadness), “*rasa bersalah*” (guilty) and “*gangguan tidur*” (sleep disturbances). The Cronbach’s alpha for BDI-M in the present study was 0.88.

### Instrumentation of musculoskeletal disorder (MSD)

The prevalence of MSD was assessed using a question that asked participants if they had experienced discomfort toward the end of their work day in the past 6 months, with a yes or no response [59]. The Cornell Musculoskeletal Disorder Questionnaire (CMSD) was used to assess the level of musculoskeletal discomfort. The CMSD is a 54-item questionnaire containing a body map diagram and questions about the prevalence of musculoskeletal ache, pain or discomfort in 18 regions of the body during the previous week. The Cronbach’s alpha for CMSD was 0.94 [60] and the reliability of CMSD in the present study was 0.97, which is very reliable.

## Data collection

Prior to data collection, an application for permission to conduct the present study at potential schools was submitted to the Ministry of Education and the State Department of Education. The researchers also contacted the potential schools and requested permission from the each of their school principals. The discussion with each school principal included setting the date and time for data collection. Each respondent signed an informed consent and received a questionnaire with a corresponding information sheet. Once they agreed to participate and the study was explained, the respondents filled out the questionnaire. Permission was given by the Malaysian Ministry of Education, the State Education Department and the school principals. Ethical approval was given by the Human Ethics Committee, Universiti Kebangsaan Malaysia.

## Data analysis

The Statistical Package for Social Science (SPSS), version 23 was used to code and analyze the data, by applying Structural Equation Modelling (SEM) with Analysis of a Moment Structure (AMOS) version 24. The data analysis begun with the data screening using exploratory data analysis (EDA) to help in detecting errors, identifying outliers, and checking assumptions on the normality of the distribution. The statistics that were used in EDA included skewness, kurtosis, boxplot, Q-Q plot, and homogeneity of variance.

The descriptive and inferential statistics were computed in the analyses on the study objectives as outlined earlier. The descriptive statistics revealed the basic distributional characteristics of all the study variables. Exploratory Factor Analysis (EFA) was used to explore the interrelationships among a set of items at the beginning of the pilot test [61]. In addition, the researchers calculated the prevalence of MSD by using SPSS. Further, the magnitude and strength of the relationship between psychosocial factors, depression and MSD were assessed using the Pearson Product-Moment Correlation analysis. Finally, path analysis was employed to determine the strength of the path shown in the path diagrams [62].

In order to test mediation, the Baron and Kenny [63] principle was used, which lists five assumptions that one must consider in establishing mediation. The first three require a significant relationship between 1) the independent variable and the mediator, 2) the independent and dependent variables, and 3) the mediator and the dependent variable. The fourth assumption is that the mediator controls the independent variable, thus affecting the outcome. The last assumption, which involves a complete mediation, is that the independent variable will have no effect on the mediator. James and Brett [64] held that in a complete mediation, the third assumption would mean no control by the mediator on the dependent variable, whereas if there is a partial mediator, the fourth assumption should be ignored [63] Thus, Pearson correlation was used to meet the first, second and third assumptions, and path analysis for the fourth and fifth assumptions. Full mediation exists when the mediator is present but the direct effect is not significant, and partial mediation exists when the direct effect is significant, but less so [62]. To determine the goodness of fit for the teachers’ MSD, the root mean square error of approximation (RMSEA) was used, with a good fit established as a value of <.06 and a comparative fit index (CFI) of > = .95 [65].

## Results

### Prevalence of MSD among teachers

In response to the question asking participants if they had experienced discomfort toward the end of the work day; a 6 month point prevalence of MSD was 80.1% (95% CI: 75.8–84.2%) with 80.5% of female and 77.5% of male teachers reporting discomfort in the preceding 6 months. Table 2 shows the frequency of MSD based on the body regions. The most experience MSD amongst participants was on their wrist (93.2%), followed by thigh (91.8%), upper arm (91.3%) and lower leg (90.5%).

**Table 2:** MSD based on body regions

Body Part	Percentage (%)
Neck	75.5%
Shoulder	80.1%
Upper back	56.4%
Upper Arm	91.3%
Lower back	59.9%
Forearm	89.6%
Wrist	93.2%
Hip/buttocks	40.9%
Thigh	91.8%
Knee	88%
Lower leg	90.5%
Foot	87.7%

### Relationships between psychosocial factor, depression and musculoskeletal disorder

There were significant relationships between psychosocial factor, depression, and MSD (see Table 3).

**Table 3:** Correlates of psychosocial factor, depression and musculoskeletal disorder

Variable	Depression	Musculoskeletal Disorder
Psychosocial Factor	−0.25^a^	−0.17^a^
Depression	–	0.30^a^

^a^Correlation is significant at the 0.01 level (2-tailed)

### Mediating effect of depression on the relationship between psychosocial factor and musculoskeletal disorder

**Fig. 2:**
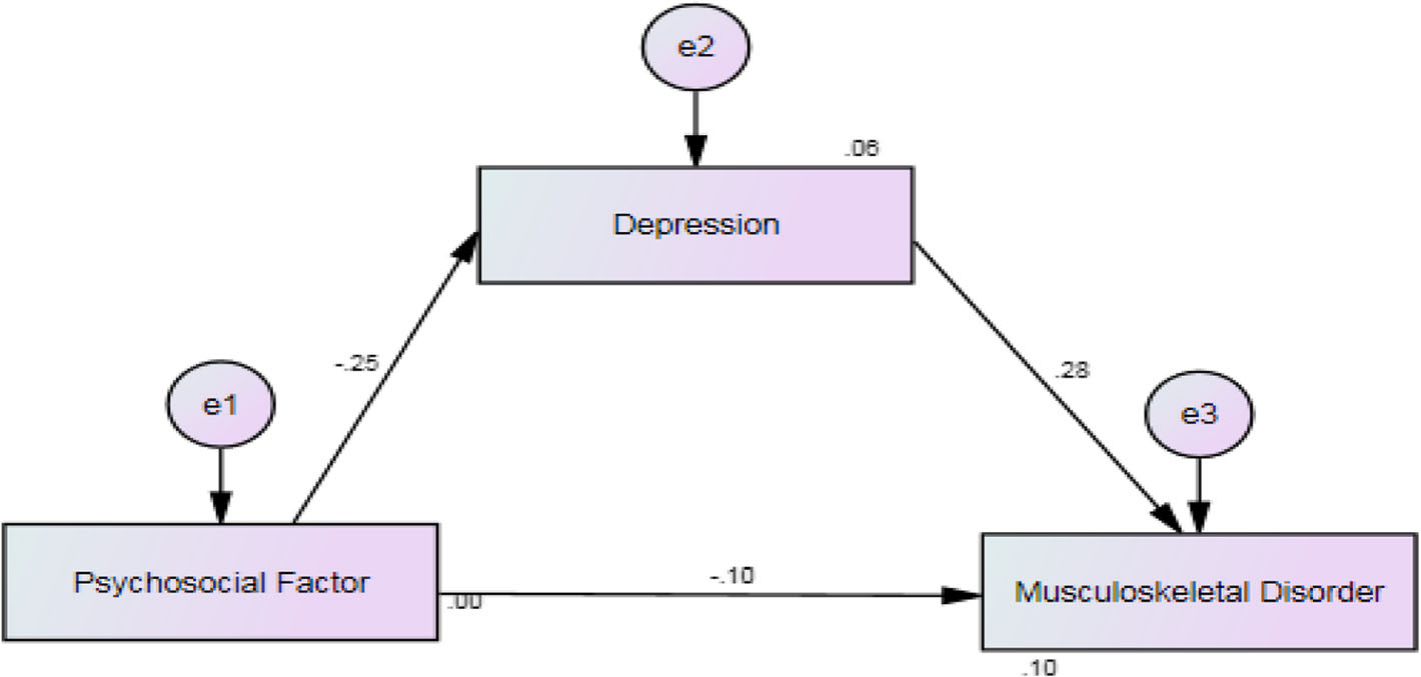
Path Analysis for the Path Model of Musculoskeletal Disorder among school teachers

When a direct path from the psychosocial factors to MSD was fixed to zero, the data were found to be acceptably fit to the model (RMSEA = .023; CFI = .990; *p* = .03). As illustrated in Fig. 2, the squared multiple correlation (R2) for depression and MSD were .06 and .10, respectively. This value indicates that 6% of the variability in depression can be explained by psychosocial factor whereas psychosocial factor and depression explained 10% of the variability in MSD.

Table 4 shows that the direct effect from psychosocial factor to MSD was statistically significant at .01, while the direct effect from depression to MSD also statistically significant at .01. Given this, the results support the alternative hypotheses stating that psychosocial factor has a direct effect on MSD and depression has a direct effect on MSD. The indirect effect of psychosocial factor to MSD through depression was −.069. The indirect effect was statistically significant when non-zero confidence interval exist. Hence, result indicates that depression was a partial mediator as the significant direct effect from psychosocial factor to MSD is reduced after the presence of depression. The result support the alternative hypothesis stating that psychosocial factor has an indirect effect on MSD through depression.

**Table 4:** Standardized direct and indirect effects on musculoskeletal disorder

Determinant	Standardized Regression coefficient	Standard error	Confidence Interval (95%)
Direct effects			
Psychosocial Factor	−.101^a^		
Depression	−.247^b^		
Indirect effects			
Psychosocial Factor	−.069^b^	.022	−.115 -.032^a^

^a^Significant level at .01

^b^Significant level at .05

## Discussion

The first aim of this study was to determine the 6-month prevalence of MSD among school teachers in Kuala Lumpur. The prevalece of MSD was 80.1% (95% CI: 75.8–84.2%), with 80.5% of female and 77.5% of male teachers reported discomfort in the past 6 months which have found a same result in a 2003 study [66] among music teachers in Sweden (82 and 80%). This prevalence was similar to another study conducted in 2014 [2] among primary and secondary school teachers in Bostwana (83.3%), and Mohseni-Bandpei and colleagues [14] study that found female teachers seemed to be more affected than their male counterpart. However, this prevalence was relatively higher when compared to other studies that have been conducted worldwide among school teachers [1, 17, 26, 27]. According to Chong and Chan [29], there was a higher incidence of MSD among primary and secondary school teachers in China (95.1%).

The prevalence rate of MSD among teachers in this present study was consistent with the findings of previous studies undertaken in Malaysia. For example, the prevalence rate in developing countries, such as Malaysian studies on MSD among primary school teachers, has been found to range between 40.4 and 74.5% [6, 7, 32, 33]. In another Malaysian study [7], the authors found that about 40.4% of the total respondents reported experiencing musculoskeletal pain during the length of service in their school. In this study, female teachers showed a significantly higher prevalence of low back pain (48.1%) than men (39.6%). A study [5] found that apart from a job as a teacher, 92.8% of the staff of the Malaysian office had experienced musculoskeletal disorders during the prior 6 months.

Many studies have been undertaken over the past 30 years in order to answer the question, “Do psychosocial factors at work cause MSD?” However, there have been many conflicting results. For example, one systematic review [55] disagreed in its conclusion regarding tthe contribution of psychosocial factors and MSD. In addition, studies [9, 10] found those psychosocial factors in the workplace such as repeated work, the burden of work and time pressure to be related to the musculoskeletal symptoms, especially in the body part of lower back as well as neck. Hoogendoorn and colleagues [10] found strong evidence showing that the risk factors for musculoskeletal disorders included low social support at work and low satisfaction among workers.

In addition, low control on the job and lack of social support by colleagues are positively associated with musculoskeletal disease. One study [67] found that nurses who reported feeling depression or stress at the beginning of the study were more likely to experience neck or shoulder pain later. In addition, tension which caused by anxiety and/or depression can increased the risk of experiencing in muscle tension and pain, change in the blood flow and oxygen supply, causing an increase in algesic substances in muscles, especially for those patients who suffering muscle pain in a long period of time. The relationship between psychosocial factors, depression and MSD is supported by previous studies in which chronic pain and depression often occur simultaneously, i.e. individuals who suffer from pain will have a higher risk of getting depression, and individuals who suffer from depression have increased the risk to experience pain [68]. Likewise, the opinion of those respondents was that that perceived psychosocial factors that were low might cause a person to have a high depression rate and thus develop the risk of MSD [6].

Nevertheless, the results of the study found that depression was a partial mediator in the relationship between psychosocial factors and MSD. This supported the predicted hypotheses (a) psychosocial factors is directly and negatively related to MSD (b) psychosocial factor has an indirect effect on MSD through depression. Parallels can be drawn in a study [69] finding a. significant relationship between psychosocial factors and MSD, especially in body parts such as shoulder pain, neck pain and upper back pain. Further findings found that high job demands were related to shoulder pain while low social support was associated with neck and upper back pain. Upper and lower back pain showed the same trend; however, the relationship was weak and not significant.

A second criterion is a relationship between psychosocial factors and depression. The present study found a relationship between depressive disorder and musculoskeletal complaints in the upper limbs, lower limbs, and back. This finding is supported by one previous study [70], while another study [71] found that an increased in mental health among patients with musculoskeletal disorders.

The third requirement, full mediation, is found when the direct effect becomes non-significant in the presence of the indirect effect, and if the direct effect is less, but still significant, partial mediation exists. The present study found that psychosocial factors and MSD were both significantly reduced after the presence of depression. According to Bair, Wu, Damush, Sutherland, and Kroenke [72], musculoskeletal pain is much more disabling when depression is present. Several studies and reviews have assessed the impact of depression on MSD/pain [6, 7, 24, 33, 36]. They agreed that the co-occurrence between MSD and depression in which individual with pain are at increased risk for depression [72, 73]. Depression is associated with pain sites, greater pain intensity, longer duration of pain, and greater likelihood of poor treatment response in those patients with pain [72]. In addition, social problems and disruption of work related with comorbid pain and depression were also reported in the literature. For example, comorbid pain and depression have reached up to 25% among patients in the Clinic of Neurology [74]. In addition, there was continuous basic of depression and pain for most of the patients after 12 months follow-up. The authors also found that baseline pain severity and the degree of depression improvement were the most influential factor in the severity of pain from time to time.

Given this, it can be suggested that depression could be one of the predictors that associated with MSD among those in the teaching profession [6, 7, 12, 17, 33, 35, 36, 75]. However, the present study was the first study to examine this relationship specifically in Malaysia.

## Strengths and limitations

The present study is one of the few studies that has examined the prevalence and predictors associated with MSD among those in the teaching profession, particularly in developing countries such as Malaysia. However, the present study also has several limitations. Firtsly**,** all variables were assessed using self-report measures, in which a general negativistic opinion towards the work situation and health status might have influenced the results. Also, the reports were only from the teachers’ views so it may not have beeb an accurate measure of the construct.

Secondly, depression was assessed with self-report measures without any further clinical interviews or assessments to diagnose specific mental disorders. Lastly, the cross-sectional nature of the analyses limits the causal inferences regarding the relationship between psychosocial factors, depression and musculoskeleal disorder. However, the study findings are in line with the latest literature and suggest that the relationship between psychosocial factors, depression and MSD is robust.

## Conclusions

Parallels can be drawn to the literature review: the findings from the present study support the idea that psychosocial factors and depression are significant predictors of MSD among teachers. MSD is much more disabling when depression is present because of the co-occurrence between MSD and depression in which individuals with pain are at increased risk for depression. Recommendations for future studies are based on the contributions and limitations as previously outlined. First and foremost is that longitudinal studies are necessary to be able to draw firm conclusions about the causal relationships between predictors and MSD. Such studies would enable greater exploration of the relationship between other potential predictors and MSD. Secondly, understanding this relationship is valuable and will assist teachers in in arranging, planning or actualizing preventive intervention programs in order to reduce the risk of MSD. This study also provides awareness for teachers and those parties involved such as the Malaysian Ministry of Education regarding the issues of MSD at the workplace. Currently, procedures and guidelines on good ergonomic movements for industrial workers involved with manual labor are readily available but not for teachers. Detailed and specific guidelines on good ergonomics for teachers are worth developing with the aim to minimize the prevalence and effects of MSD among teachers. Third, future intervention studies on how to reduce MSD among teachers is therefore warranted. In a nutshell, the study, within the limits posed by its cross-sectional design, supported the hypothesis that depression partially mediates the effects of psychosocial work conditions on MSD. Given this, preventive measures for MSD should also take into account these two important predictors, that is psychosocial factors and depression, in order to minimize the impact of MSD among those in the teaching profession.

## Abbreviations

AMOS: Analysis moment structures
EDA: Exploratory data analysis
EFA: Exploratory factor analysis
MSD: Musculoskeletal disorder
SEM: Structural equation modelling
SPSS: Statistical package for social science
